# The commonly-used DNA probe for diffusely-adherent *Escherichia coli *cross-reacts with a subset of enteroaggregative *E. coli*

**DOI:** 10.1186/1471-2180-9-269

**Published:** 2009-12-21

**Authors:** Anna M Snelling, Louissa R Macfarlane-Smith, Jonathan N Fletcher, Iruka N Okeke

**Affiliations:** 1 Division of Biomedical Sciences and Bradford Infection Group, University of Bradford, Bradford, West Yorkshire, BD7 1DP, UK; 2 Department of Biology, Haverford College, 370 Lancaster Avenue, Haverford, PA 19041, USA

## Abstract

**Background:**

The roles of diffusely-adherent *Escherichia coli *(DAEC) and enteroaggregative *E. coli *(EAEC) in disease are not well understood, in part because of the limitations of diagnostic tests for each of these categories of diarrhoea-causing *E. coli*. A HEp-2 adherence assay is the Gold Standard for detecting both EAEC and DAEC but DNA probes with limited sensitivity are also employed.

**Results:**

We demonstrate that the *daaC *probe, conventionally used to detect DAEC, cross-reacts with a subset of strains belonging to the EAEC category. The cross hybridization is due to 84% identity, at the nucleotide level, between the *daaC *locus and the aggregative adherence fimbriae II cluster gene, *aafC*, present in some EAEC strains. Because *aaf*-positive EAEC show a better association with diarrhoea than other EAEC, this specific cross-hybridization may have contributed to an over-estimation of the association of *daaC *with disease in some studies. We have developed a discriminatory PCR-RFLP protocol to delineate EAEC strains detected by the *daaC *probe in molecular epidemiological studies.

**Conclusions:**

A PCR-RFLP protocol described herein can be used to identify *aaf*-positive EAEC and *daaC*-positive DAEC and to delineate these two types of diarrhoeagenic *E. coli*, which both react with the *daaC *probe. This should help to improve current understanding and future investigations of DAEC and EAEC epidemiology.

## Background

Enteropathogenic, enterotoxigenic, enteroinvasive, enterohaemorrhagic and enteroaggregative *Escherichia coli *are categories of enteric *E. coli *that have been unequivocally associated with diarrhoeal disease through human challenge studies and/or outbreak investigations [1]. Regarding other potentially diarrhoeagenic categories of *E. coli*, the most evidence for enterovirulence has been compiled for diffusely adherent *E. coli *(DAEC). However, the basis for DAEC pathogenicity is not well understood. The category is heterogeneous and although some studies have shown an association of DAEC with diarrhoea, others have not [2]. Two DAEC strains did not elicit diarrhoea upon human volunteer challenge and no outbreaks of DAEC-associated illness have been documented to date [3].

Enteroaggregative *E. coli *(EAEC) is another heterogeneous diarrhoeagenic *E. coli *category. Convincing epidemiological information from EAEC outbreaks exists, and at least one strain was diarrhoeagenic in some human volunteers, however the category is very diverse (reviewed in references [4] and [5]). Compared to other diarrhoeagenic *E. coli *categories, EAEC and DAEC pathotypes were both described relatively recently and their epidemiology, risk factors and pathogenesis are still in early stages of investigation. Few epidemiological studies seek these categories because the Gold Standard test for their detection, the HEp-2 adherence assay, is cumbersome. This tissue culture-based test requires expensive facilities and technical expertise that are not universally available.

An improved understanding of the importance of diarrhoeagenic *E. coli *in human disease will depend upon reliable epidemiological data and on channelling of strains identified into molecular pathogenesis research. Accordingly, efforts have been made to develop more widely applicable methods to detect EAEC and DAEC. Baudry *et al*. tested fragments from the large plasmid of EAEC strain 17-2 and identified a 1 Kb fragment, CVD432, which was 89% sensitive and 99% specific for EAEC strains in their collection [6]. Subsequently, this probe has continued to show specificity for EAEC but its sensitivity has varied between 15 and 90% in different studies [4]. Bilge *et al*. [7] used a different approach to generate a diagnostic probe for DAEC. They identified, cloned and characterized the F1845 adhesin from DAEC strain C1845. The F1845 adhesin belongs to the Afa/Dr family and is encoded by a five-gene cluster [2]. Bilge *et al*. [7] proposed part of the *daaC *gene of the encoding operon as a marker for DAEC strains. From the time of its discovery, it has been known that the cloned *daaC *fragment probe (in plasmid pSLM862) can only identify a subset of DAEC and that some DAEC strains have other adhesins, of which many, but not all, are from the Afa/Dr family [2]. However, the *daaC *probe is the one that has been employed most frequently in epidemiological research to date 8-13. In this paper, we report that the *daaC *cross-hybridizes with a specific subset of EAEC strains. We sought to identify the molecular basis for this cross-hybridization and to devise an alternate, cost-effective protocol for identifying DAEC.

## Methods

### Strains

Cross reaction of the *daaC *probe with EAEC was identified in the course of screening 509 test *E. coli *strains, which were isolated from 130 travellers with diarrhoea (up to four isolates were obtained from each specimen), who returned to the UK in 2002-2003, from a total of 33 different countries [14]. We additionally employed 26 well-characterized archival EAEC strains and seven DAEC strains for control purposes. *E. coli *K-12 TOP-10 (Invitrogen) was used to maintain plasmids and non-pathogenic strains DH5α and MG1655 were used as non-adherent controls.

### Routine molecular biology procedures

Standard molecular biology procedures were employed [15]. DNA amplification was performed using 1 unit recombinant *Taq *polymerase enzyme, 2 mM magnesium chloride, PCR buffer (Invitrogen, Carlsbad, CA) and 1 μM oligonucleotide primer in each reaction. All PCR amplifications began with a two-minute hot start at 94°C followed by 30 cycles of denaturing at 94°C for 30s, annealing for 30s at 5°C below primer annealing temperature and extending at 72°C for 1 minute for every Kb of DNA being amplified. PCR reactions were templated with boiled bacterial colonies or genomic DNA. High fidelity PCR for sequencing used a similar protocol but employed *Pfx *polymerase and magnesium sulphate (Invitrogen). The annealing temperature was lowered by 2-3°C and extension time was doubled for *Pfx *high-fidelity PCR. Purified PCR-amplified fragments were incubated with *Taq *polymerase and dNTPs at 72°C for 20 minutes and then cloned into the pGEM-T vector (Promega) according to manufacturer's instructions. Plasmids were transformed into chemically competent *E. coli *K-12 TOP10 cells (Invitrogen).

### Colony hybridization

Colony lifts of test and control strains cultured in Brain Heart Infusion medium (Oxoid, England) were prepared in a 96-well format on nylon membrane (Hybond-N, Amersham Biosciences). The membranes were denatured in 0.5 M NaOH, 1.5 M NaCl, neutralized in 1.5 M NaCl, 0.5 M Tris HCl and 1 mM EDTA, dried and fixed by UV exposure. DNA probes consisted of PCR products using the primers in Table 1. The probes were labelled using the PCR DIG labelling mix (Roche), according to manufacturer's instructions. Cloned probes were labelled using M13F and M13R universal primers. The vector-derived ends of the probe were then excised with specific restriction endonucleases and the labelled probe purified. Following 2 hours pre-hybridization at 42°C, the membranes were hybridized with denatured probe at 42°C, with continuous, gentle agitation in a hybridization solution containing 50% formamide, 5X SSC, 5% blocking reagent, 0.1% N-lauryl sarcosine and 0.02% SDS. The membranes were washed three times in 2X SSC, 0.1% SDS and then three times in 0.1% SSC, 0.1% SDS. Signal was detected using the DIG nucleic acid detection kit (Roche) in accordance with manufacturer's instructions.

**Table 1:** Oligonucleotides used in this study

Primer designation	oligonucleotides	Target/application	Predicted product	Reference/source
CVD432F	5'-CTG GCG AAA GAC TGT ATC AT-3'	AA probe (CVD 432)	629 bp	[43]
			
CVD432R	5'-CAA TGT ATA GAA ATC CGC TGT T-3'			

aapF	5'-CTT GGG TAT CAG CCT GAA TG-3'	*aap*, encoding the enteroaggregative *E. coli *plasmid-borne anti-aggregation protein, dispersin	310 bp	[44]
			
aapR	5'-AAC CCA TTC GGT TAG AGC AC-3'			

aggAF	5'-TTA GTC TTC TAT CTA GGG-3'	*aggA*, encoding the structural subunit of aggregative adherence fimbriae I	450 bp	[17]
			
aggAR	5'-AAA TTA ATT CCG GCA TGG-3'			

aggRF	5'-CTA ATT GTA CAA TCG ATG TA-3'	*aggR*, encoding the enteroaggregative *E. coli *plasmid-borne aggregative adherence regulator	457 bp	[44]
			
aggRR	5'-AGA GTC CAT CTC TTT GAT AAG-3'			

M13F	5'-GGT TTT CCC AGT CAC GAC-3'	Vector priming sequencing primer	Not applicable	

M13R	5'-CAG GAA ACA GCT ATG ACC-3'	Vector priming sequencing primer	Not applicable	

aafBdaaDF	5'-CCTGCGGGATGTTACT-3'	*aafB *from EAEC and *daaD *from DAEC	333/339	This study
			
aafBdaaDR	5'-GCCATCACATCAAAAA-3'			

### HEp-2 adherence assay

HEp-2 adherence tests were performed as described by Vial *et al*. [16]. Bacteria were cultured in LB broth without shaking at 37°C overnight. HEp-2 cell monolayers were cultured overnight in 8-well chamber slides to 50% confluence in high glucose DMEM with foetal bovine serum, streptomycin and penicillin (Invitrogen) and then washed three times with PBS. 300 μL of high-glucose DMEM media containing 1% mannose (without foetal bovine serum and antibiotics) and 10 μL of bacterial culture was added to each chamber. After 3h incubation, the media was aspirated and the monolayer washed three times with PBS. The cells were fixed for 20 minutes with 70% methanol and then stained for 20 minutes with a 1:40 dilution of Giemsa in PBS. Adherence patterns were observed using oil immersion light microscopy at 1000x magnification. All bacterial isolates were tested in duplicate and replicates were read by two different individuals.

### Sequence analyses

The EAEC 042 genome sequence was accessed from *Escherichia coli *and *Shigella *spp. comparative Sequencing Group at the Sanger Institute, and can be accessed at http://www.sanger.ac.uk/Projects/Escherichia_Shigella/. All other sequences were retrieved from GenBank. The 042 *daaC *cross-hybridizing region was identified by nucleotide BLAST, employing a BLOSUM62 matrix with a low complexity filter. Pair-wise alignments and computations of % identity were done using FASTA and multiple alignments were generated using CLUSTAL.

### PCR-RFLP

We devised a PCR-Restriction Fragment Length Polymorphism (PCR-RFLP) test for *daaD/afaD *and *aafB*. Using primers aafBdaaDF and aafBdaaDR, which are complementary to regions conserved between the two targets, we amplified a 333 bp (*daaD*) or 339 bp (*aafB*) PCR product. Recombinant *Taq *polymerase enzyme and PCR buffer from NEB were employed with 1 unit of *Taq *polymerase, 2 mM MgCl_2 _and 1 μM oligonucleotide primer in each reaction. We additionally repeated 48 amplifications using PCR-Supermix (Invitrogen) and obtained identical results. All amplifications began with a two-minute hot start at 94°C followed by 35 cycles of denaturing at 94°C for 30s, annealing at 41°C for 30s at and extending at 72°C for 20s. PCR reactions were templated with boiled bacterial colonies or genomic DNA. Strains containing the *daaD *or *aafB *gene gave a predicted 333 or 339 bp product respectively. This product was digested with the restriction enzyme *Alu*I. The digestion generates two predicted fragments for *aafB *and five fragments for the more GC rich *daaD *gene, which can be resolved on a 2% TBE agarose gel.

## Results

### The *daaC *probe cross-hybridizes with a sub-set of EAEC

**Figure 1:**
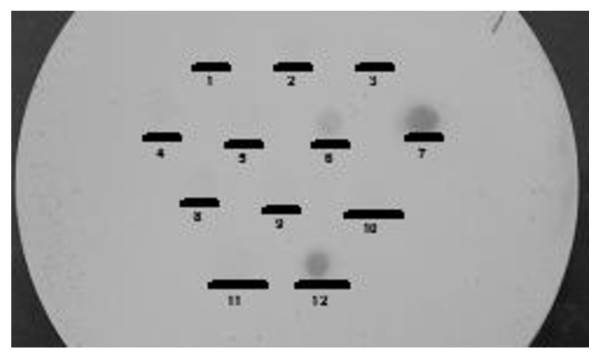
**Colony blot of representative reference strains hybridized to the *daaC *probe from pSLM862**. The blot was hybridized with biotinylated probe under high stringency conditions. Strains spotted on the membrane were 1: *E. coli *K-12 DH1, 2: *E. coli *NCTC 10418, 3: enteropathogenic *E. coli *E2348/69, 4: enterohaemorrhagic *E. coli *C412, 5: enterotoxigenic *E. coli *H10407, 6: enteroaggregative *E. coli *042, 7: diffusely adherent *E. coli *DAEC1, 8: enterotoxigenic *E. coli *P006413, 9: enterotoxigenic *E. coli *P006371, 10: enteroinvasive *E. coli *G24; 11: Cytotoxic necrotizing toxin-producing *E. coli *P006254, 12: diffusely-adherent *E. coli *DA57-1166.

In the course of an aetiologic study of diarrhoea focused on diarrhoeagenic *E. coli*, we observed that in addition to recognizing diffusely adherent *E. coli *strains, the *daaC *probe was hybridizing to colony blots of some test and control strains that showed aggregative adherence. We hybridized the *daaC *probe with colony blots of a well-studied panel of 26 EAEC strains and seven DAEC strains. We found that five of these EAEC strains hybridized with the *daaC *probe, including prototypical EAEC strain 042, even when conditions were of slightly greater stringency than those reported in the literature [11]. All five had previously been documented to carry the *aafA *gene, encoding the structural subunit of the AAF/II fimbriae [17]. As shown in Figure 1, hybridization was noticeably weaker than to the DAEC strains, but sufficiently strong to confound strain categorization. Twenty-one strains lacking *aafA *did not hybridize with the *daaC *probe, irrespective of whether they hybridized to the probe for *aggA*, the structural subunit gene for AAF/I fimbriae (Table 2).

**Table 2:** Hybridization of well-studied EAEC and DAEC strains to EAEC probes and *daaC *and results of PCR-RFLP test for *daaD *and *aafB*.

**Strain**	**Serotype**	**Country of isolation/source**	HEp-2 adherence pattern*	**pAA (CVD 432)**	***aap***	***aggA***	***aafA***	***daaC*** **hybrid-ization (SLM 862)**	***aafB/daaD *RFLP**
AA 60A		Mexico	Aggregative	+	+	+	-	-	-
AA H232-1		Peru	Aggregative-detaching	+	+	-	-	-	-
AA 17-2	O3:H2	Chile	Aggregative-detaching	+	+	+	-	-	-
AA 253-1	O3:H2	Thailand	Aggregative	+	+	+	-	-	-
AA 6-1	OR:H2	Thailand	Aggregative	+	+	-	-	-	-
BM369	O86	India	Aggregative	-	-	-	-	-	-
AADS65-R2		Philippines	Weak localised-aggregative	-	-	-	-	-	-
AA 501-1	OR:H53	Thailand	Aggregative	-	-	-	-	-	-
AA H223-1		Peru	Aggregative	+	+	-	-	-	-
AA DS67-R2		Philippines	Aggregative	+	+	+	-	-	-
AA 042	O44:H18	Peru	Aggregative	+	+	-	+	+	*aafB*
AA 144-1	O77:NM	Thailand	Aggregative-detaching	+	+	-	-	+	*aafB*
AA 44-1	O36:H18	Thailand	Aggregative	+	+	-	-	-	-
AA H145-1		Peru	Aggregative	+	+	+	-	-	-
AA 309-1	O130:H27	Thailand	Aggregative	+	+	+	-	+	*aafB*
H133		Peru	Aggregative	+	-	-	-	-	-
MH46-2		Peru	Aggregative	+	+	-	-	-	-
M32-1		Peru	Aggregative	-	+	-	-	-	-
C04		Nigeria	Aggregative	+	+	-	-	-	-
C08		Nigeria	Aggregative	+	+	-	-	-	-
AA 103-1	O148:H28	Thailand	Aggregative	+	+	-	-	-	-
AA 435-1	O33:H16	Thailand	Aggregative	+	+	-	+	+	*aafB*
AA 199-1	OR:H1	Thailand	Aggregative	+	+	-	+	+	*aafB*
AA H194-2		Peru	Aggregative	+	+	+	-	-	-
AA 278-1	O125ac:H21	Thailand	Aggregative	+	+	-	-	-	-
AA 239-1	OR:H21	Thailand	Aggregative	+	-	-	-	-	-
AA 101-1	O?:H10	Japan	Aggregative	-	-	-	+	+	aafB
G02a		Nigeria	Aggregative-detaching	-	-	+	-	-	-
D163		UK	Aggregative	-	+	-	-	-	-
AA H92-1		Peru	Aggregative	-	-	-	-	-	-
DH5α		CVD**^†^**	Non-adherent	-	-	-	-	-	-
MG1655		CVD**^†^**	Non-adherent	-	-	-	-	-	-
DAEC1		CVD**^†^**	Diffuse	-	-	-	-	+	*daaD2*
DA57-1186		CVD**^†^**	Diffuse	-	-	-	-	+	*daaD*
DA55-2186		CVD**^†^**	Diffuse	-	-	-	-	+	*daaD*
DAEC4		CVD**^†^**	Diffuse	-	-	-	-	+	*daaD*
TW6350	O157:H45	T. Whittam	Diffuse	-	-	-	-	+	*daaD2*
DAWC21211	?O81:NM	Thailand	Diffuse	-	-	-	-	-	-

* HEp-2 adherence patterns determined by the 3h assay described by Vial *et al*. [16]

^†^CVD = Center for Vaccine Development stocks, University of Maryland, Courtesy of JP Nataro

From a second, and larger, collection of 509 test *E. coli *strains from 130 recent travellers with diarrhoea, 48 showed aggregative adherence (AA), 52 diffuse adherence (DA), and 181 were non-adherent [14] (Table 3). Another 228 showed some degree of adherence, ranging from very weak diffuse to strong but indeterminate patterns of adherence. These included 49 strains with a pattern that had elements of both aggregative and diffuse adherence, termed AA/DA. The *daaC *probe hybridized with only 2 (1.1%) of the non-adherent strains and with 60 (18.3%) adherent bacterial isolates. Of these, 28 were diffusely-adherent, nine displayed aggregative adherence, 22 showed AA/DA and the remaining strain had cell-detaching properties. Although the sensitivity of the *daaC *probe for DAEC or DAEC plus AA/DA strains combined was low (53.8 and 49.5% respectively), as has been previously acknowledged, the specificity and positive predictive value for DAEC were considerably higher (at 93.2 and 45.2% respectively). These rates are comparable or better than values for other probes for aggregative or diffusely adherent *E. coli*. However the false positives identified by the *daaC *probe were not randomly distributed across *E. coli *categories. The *daaC *probe recognized 18.8% (9 out of 48) of aggregative adherent strains but only 1.1% of non-adherent strains (Table 3, p < 0.0001; Fishers exact test).

**Table 3:** Adherence patterns of 509 isolates collected prospectively from 130 travellers with diarrhoea and their hybridization to the *daaC *probe.

**Adherence pattern**	**Number of isolates showing pattern (n = 509)**	**Number (%) of isolates hybridizing to the *daaC *probe**
AA	48	9 (18.8)
DA	52	28 (53.8)
AA/DA	49	22 (44.9)
Other adherence patterns (non AA or DA)	179	1 (0.6)
Non-adherent	181	2 (1.1)

AA = aggregative adherence; DA = diffuse adherence; AA/DA = elements of both aggregative and diffuse adherence

To verify that the hybridizing aggregative adherent strains were true and typical EAEC, that is strains carrying a partially conserved plasmid referred to as pAA, we screened them for EAEC virulence loci. Only one of the nine aggregative adherent *daaC*-positive strains hybridized with the CVD432 probe [6], but seven of the nine strains hybridized with at least one other EAEC probe (the pAA-borne *aggC *for aggregative adherence fimbrial usher [18] or *aap *for dispersin [19] or the chromosomal gene *pic *for mucinase, which is also present in *Shigella *[20]). Only one *daaC*-positive strain showing aggregative adherence did not hybridize with one of the four EAEC probes we employed. Importantly, all but one of nine *aafA-*positive EAEC strains identified among the 509 *E. coli *isolates hybridized with the *daaC *probe. Four of the nine *daaC*-positive EAEC strains were from the same individual and probably clonal. The other five were from five separate patients, who were recent returnees from four different countries. Overall, evidence from two independently derived strain sets suggests that the *daaC *probe recognizes a specific subset of EAEC, that is strains that possess *aafA*.

### The *daaC *cross-hybridizing locus in EAEC is *aafC*

The *daaC *probe is excised from plasmid pSLM862 with *Pst*I prior to use (7). We used vector-priming M13 oligonucleotides to sequence the pSLM862 insert, which we have deposited in the Genbank database (Accession Number EU010379). A BLAST search of the Genbank nucleotide database revealed that the *daaC *probe was 97% identical to *draC*/*afaC*/*dafaC *genes from other, diffuse-adherence associated operons in the Genebank database (Accession numbers AF325672.1, X76688.1 and AF329316.1).

**Figure 2:**
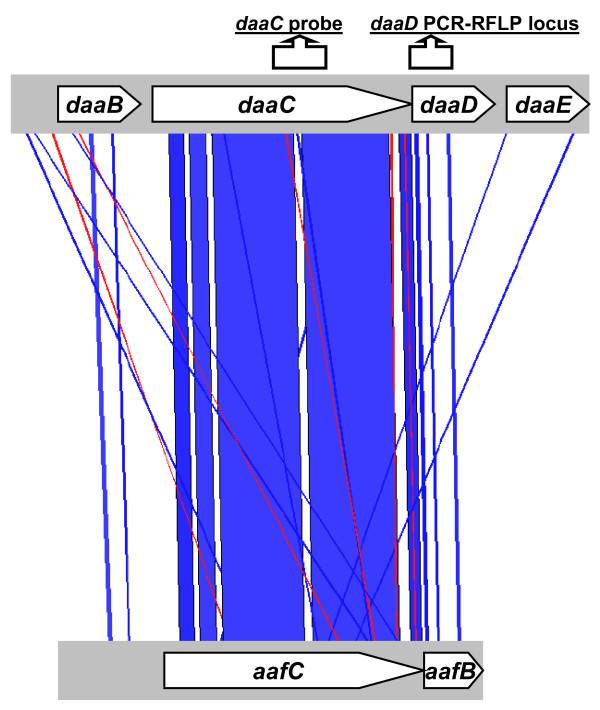
**BLAST alignment of a diffuse adherence *dafa/daa *operon (Accession number AF325672) and region 2 of the *aaf*/II operon from strain 042 (Accession number AF114828)**. Genbank Annotated orfs are shown for *dafa *(top) and *aaf*, region 2 (bottom). Connectors show regions of 80% or more identity at the DNA level. The figure was generated using the Artemis Comparison Tool (ACT)[45].

A BLAST search of the recently completed genome of cross-hybridizing EAEC strain 042 at http://www.sanger.ac.uk/cgi-bin/blast/submitblast/escherichia_shigella, revealed that the most similar target for the *daaC *probe that can be identified in the 042 genome *in silico *is the *aafC *gene, part of the AAF/II-encoding operon, with 294 (84%) identical nucleotides and only five single nucleotide gaps over the length of the homologous 344 nucleotide *daaC *probe region, at the DNA level (Figure 2). The *aafC *gene is located on the large virulence plasmid of strain 042 and other AAF/II-positive EAEC [21]. The *daaC *gene, on the other hand, may be chromosomally or plasmid located [7]. Therefore, although genuine target strains often have only one copy of *daaC*, cross hybridizing strains could potentially have one or more copies of the *aafC *gene, a factor that could also contribute to the hybridization signals of *aafC*-positive EAEC. Elias *et al*. have previously noticed that enteroaggregative *E. coli *strains hybridize to the *daaC *probe and proposed that the cross-hybridizing region was within the AAF/II fimbrial biogenesis cluster [21]. In this study, all but one strain possessing the *aafA *gene from the AAF/II biogenesis cluster hybridized with the *daaC *probe. We hybridized the panel of 26 well-studied strains to a DNA fragment probe for the aggregative adherence fimbrial usher gene, *aggC*, which has been demonstrated by Bernier *et al*. to hybridize to both *aggC *and *aafC *[18]. All the *aafA*-positive, *daaC*-positive strains hybridized with this probe (Table 2). In summary, we report that *daaC *cross-hybridization arises from an 84% identity between the probe sequence and the EAEC *aafC *gene, and that this degree of similarity significantly compromises diagnostic use of the existing *daaC *probe for the detection of DAEC.

### Development of a PCR-RFLP protocol to detect and delineate *daaC *and *aaf-*positive strains

**Figure 3:**
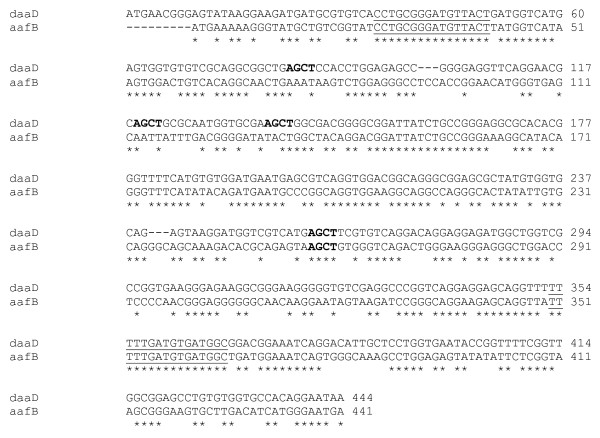
**Pair-wise alignment between the *daaD *and *aafB *gene regions used as a basis for a discriminatory PCR-RFLP**. Identities are asterixed. Oligonucleotide binding sites for the PCR-RFLP protocol are underlined and *Alu*I restriction sites are highlighted in boldface.

The *daaC, aafC *and similar genes are predicted to encode ushers for adhesin export and are highly similar across the entire length of the genes, both to each other and to usher genes from other adhesin operons (Figure 2). Downstream of the usher genes is a smaller open reading frame. In the case of the EAEC *aafC*, the downstream gene, *aafB*, has not been experimentally defined and may encode a protein that represents the AAF/II tip adhesin [22]. The *aafB *predicted product shares 59% identity with the DAEC AfaD/DaaD, a non-structural adhesin encoded by a gene downstream of *afaC/daaC *[21]. At the DNA level, *aafB *and *daaD/afaD *genes also share some identity (63% over the most similar 444 bp region), but this is less than that of the usher genes (Figure 3).

**Figure 4:**
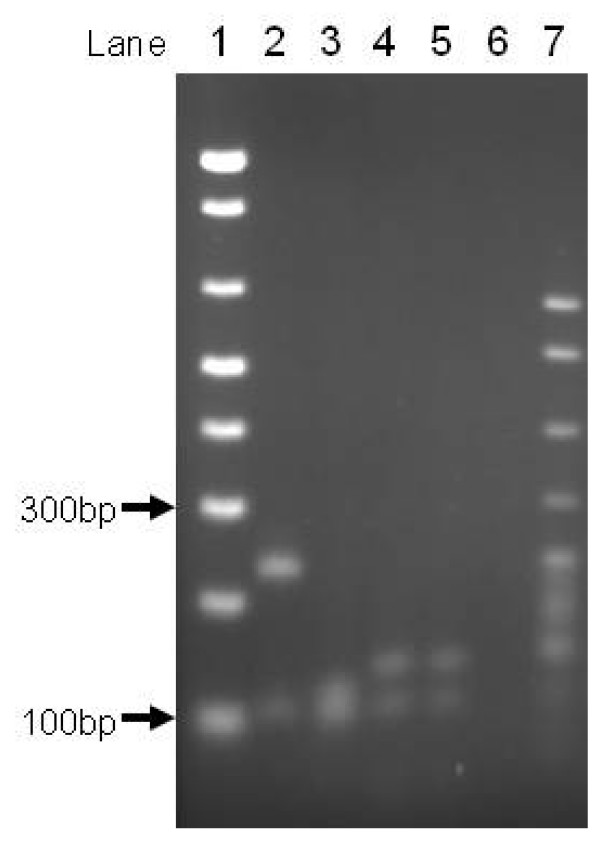
**PCR-RFLP to distinguish *daaD *and *daaD2 *from *aafB***. Lane 1: 1 Kb Ladder Plus (Invitrogen); Lanes 2-6: *Alu*I restricted amplicons from EAEC strain 042 (*aafB*), DAEC strains 1 (*daaC2*), 2 and 3 (*daaC*) and non-pathogenic strain HS. Lane 7: pBR322 *Msp*1 marker (NEB).

As shown in Figure 2, three regions of similarity between *afaD *and *aafB*, at the DNA level, are interspersed by two dissimilar regions. We devised a PCR-Restriction Fragment Length Polymorphism (PCR-RFLP) test for *daaD/afaD *and *aafB *using primers complementary to regions conserved between the two targets, and digesting the 333/339 bp product with the restriction enzyme *Alu*I. The digestion generates two fragments for *aafB *(233 and 106 bp) and five fragments for the more GC rich *daaD *gene (123, 106, 50, 36 and 18 bp). As shown in Figure 4, whilst the smallest *daaD *fragments are not visible, the two profiles are easily distinguished on a 2% agarose gel.

In the course of our investigations, we identified a third restriction profile, initially from strain DAEC1 (Figure 4). We sequenced the amplified region from this strain and determined that although the probe showed a 100% identity with *daaD *over most of its sequence, there was a 60 bp region with no significant homology. We refer to this allele as *daaD2*, and have deposited the sequence in GenBank (Accession Number EU010380). *daaD2 *lacks the two *Alu*I sites closest to the 5' end of *daaD *(Figure 2), which lie within the non-conserved region, but otherwise is very similar to *daaD*. Digestion of the PCR product from this allele yields 3 fragments of 104, 109 and 120 bp, which are irresolvable on a 2% gel but produce a profile easily distinguished from that of *aafB *and *daaD *(Figure 4). We found that *daaD *was more common than *daaD2 *in our collection. Additionally, there are four sequences from strains bearing identical or nearly identical (>99% identity) *daaD2 *alleles already deposited in GenBank [23], but as many as 20 sequences from an equivalent number of strains with classic *daaD *alleles. This does suggest that *daaD *may be the more common allele, but the epidemiological significance of the variation, if any, in these alleles is unclear.

## Discussion and conclusion

There have been brief mentions of *daaC *hybridization with EAEC in the literature. In some studies, the hybridization of the *daaC *probe to enteroaggregative *E. coli *has been taken to mean that the strains in question harbour a *daa *adhesin target as well as aggregative adherence genes [24]. Other workers have proposed that the hybridization signal arises from cross-hybridization at a single locus [21,25]. Although the former situation is a possibility, particularly as aggregative fimbrial genes are plasmid-borne, in this study we implicate the *aafC *gene, predicted to encode the usher for AAF/II fimbriae, as a cross-hybridizing locus. This finding has implications for our current understanding of the epidemiology of diarrhoeagenic *E. coli*.

Understanding the aetiology of diarrhoea is important, particularly in high disease burden areas where risk factors need to be identified and vaccine development priorities established. Most of what is known about the relative importance of different diarrhoeagenic *E. coli *categories comes from small, snapshot studies or studies of traveller's diarrhoea, analogous to what Guerrant *et al*. [26] refer to as the 'eyes of the hippopotamus'. Many high-burden developing countries lack cell culture facilities for the Gold Standard HEp-2 assay needed to delineate some pathotypes of diarrhoea-causing *E. coli *from commensals. Non-radioactive DNA probes and, more recently, PCR have been advocated as methodology that might be used to detect enterovirulent *E. coli *in developing countries [27,28]. The vast majority of earlier studies that have not used HEp-2 adherence assays have defined DAEC as *E. coli *that hybridize to the *daaC *probe.

Of 30 Medline-indexed controlled studies that sought DAEC, we were able to identify only nine that have heretofore demonstrated an association of DAEC with diarrhoea. Girón *et al*. [29] used *daaC *probe hybridization and HEp-2 adherence and found that DAEC were associated with disease in Mayan children in Mexico. However that study had a very short duration (3 weeks) and focused on a small remote population (63 cases, 1300 total population), and therefore there are limits to the extent to which the data should be extrapolated. Cegielski *et al*. [30] found probe-positive, but not diffuse-adherent DAEC associated with chronic diarrhoea in HIV-positive and HIV-negative patients in another small study in Tanzania. A recent Brazilian study made a similar finding: probe-positive DAEC were associated with paediatric diarrhoeal disease, particularly in older children [13]. A Bangladeshi study reported that DAEC identified by adherence assay were associated with persistent but not acute diarrhoea (p < 0.05)[31]. A number of other developing country studies published since that time, employing probe and adherence, adherence alone, or PCR-based detection have failed to find an association between detection of DAEC and disease [8,10,12], 32-35.

In 1993, Levine *et al*. observed that a Chilean study, entirely reliant on the *daaC *probe, represented the "strongest epidemiologic evidence so far to indicate that DAEC may indeed be pathogenic"[36]. This large, controlled cohort study identified DAEC, based on *daaC *hybridization alone, in 16.6% of cases and 11.9% of controls (p = 0.0024). In that study, children aged 4-5 years had a relative risk of 2.1 for DAEC (overall relative risk was 1.4). Subsequent reports from studies using only the probe support the findings of that study [13,37,38]. For example, a 2005 US study found that DAEC identified by SLM862 probe were associated with diarrhoea (p < 0.05) but DAEC identified by HEp-2 adherence were not [38]. Overall, in the light of the limitations of the *daaC *probe we here report, only three published studies that we reviewed unequivocally suggest a role for DAEC in acute diarrhoeal disease. Jallat *et al*. [11] used HEp-2 adherence to identify DAEC in a French study and found these organisms to be significantly associated with disease in patients of all ages (p < 0.0001). In that study, only 33 of the 100 DAEC isolates identified hybridized with the *daaC *probe and interestingly, five of these strains also hybridized with the CVD432 probe for enteroaggregative *E. coli *and showed an aggregative-diffuse pattern of adherence. Ten *daaC *positive strains were non-adherent. A second study, by Gunzburg *et al*. [39], found that DAEC were not associated with diarrhoea overall, and were more common in healthy patients under 18 months of age. However, Gunzburg *et al*. did find that in children aged 18 months to five years, DAEC were recovered from 11 cases and 4 controls (p ≤ 0.05). Similarly, Scaletsky *et al*. [9] found that DAEC was not associated with disease overall in a study performed in North-East Brazil but was significantly associated with diarrhoea among children in the 13-24 month old age group. These studies provide evidence to advocate that future investigations aim to determine whether there is a role for DAEC in diarrhoea in some populations, particularly in children over one year of age, and that they do so using techniques other than the *daaC *probe.

There are important implications for the role of pathogens other than DAEC in disease that may come to light if the *daaC *probe is replaced with more specific testing methods. Recent studies have demonstrated that AAF/II-positive EAEC are more significantly associated with diarrhoea than the EAEC category as a whole 40-43. Thus any test for DAEC that detects potentially AAF/II EAEC will skew the results towards a stronger association of the DAEC category with disease, particularly if the EAEC strains in question are negative for the commonly used but inadequately sensitive EAEC CVD432 probe. Additionally, evidence supporting a role in diarrhoea for less-studied *E. coli *categories such as cell-detaching *E. coli *or cytolethal distending toxin-producing *E. coli*, appears to be equivalent to supporting data for DAEC, if *daaC*-derived data is discounted. Future investigators may want to consider these under-studied categories as worthy of further study.

There is some suggestion that DAEC could be an important pathogen in weaned children but in order to correctly gauge the relative contributions of DAEC and other pathogens such as AAF/II-producing EAEC to diarrhoea epidemiology, it is imperative that the SLM862 *daaC *probe, which detects AAF/II-positive EAEC as well as DAEC, be discarded in favour of more specific methodology. Given that AAF/II-positive EAEC represent an important subset of that category and therefore there is considerable advantage of testing for both simultaneously, particularly as current PCR-based protocols typically do not screen for DAEC and use CVD432 as the EAEC target [28]. If the *daaC *probe is employed, it should be used in conjunction with a probe for *aafA*. Alternatively, the PCR-RFLP test we describe here, which delineates the adjacent *daaD *and *aafB *genes may be substituted for hybridization with the SLM862 cloned *daaC *probe.

## Authors' contributions

AS co-conceived the study, designed and coordinated the work, contributed to reading HEp-2 adherence assay slides, and provided significant input into writing the manuscript. LRM-S performed and read HEp-2 adherence assays, performed DNA hybridizations and maintained and mined strain databases. JNF contributed to reading HEp-2 adherence assay slides and made contributions to writing the manuscript. INO co-conceived the study, performed sequence analyses, designed and validated the PCR-RFLP and wrote the first draft of the manuscript. All authors read and approved the final manuscript.
